# A Further Inquiry Concerning Constitutional Irritation, and the Pathology of the Nervous System

**Published:** 1836-07

**Authors:** 


THE
### BRITISH AND FOREIGN
### MEDICAL REVIEW,
FOR JULY, 1836.

---

### PART FIRST.

### Analytical and Critical Reviews.

---

### Art. I.

*A further Inquiry concerning Constitutional Irritation, and the Pathology of the Nervous System.* By Benjamin Travers, F.R.S., Senior Surgeon to St. Thomas's Hospital, &c.—*London*, 1835. 8vo. pp. 444.

It is probable that practical surgeons, at all periods since surgery was cultivated as a science, have been accustomed to reflect on, and to take into account, the effect of local injuries on the body; and, on the other hand, the influence of a faulty state of the constitution on local affections. Close observers could scarcely overlook such marked and evident phænomena; and, in fact, frequent allusions are made by the older writers to these important circumstances. Thus, to take an instance from a surgeon who wrote at a time when surgery was so subordinate a branch of the healing art that he considered it necessary to warn the students of his day "not to rest satisfied with being able to shave, spread a plaster, or open a vein:" we find Heister* alluding to local injuries producing "symptomatic fever," and, on the other hand, "bad habits of body, as the scorbutic, hydropical, and consumptive," preventing the cure of wounds. To John Hunter, however, we are first indebted for having investigated the subject as a distinct and important branch of surgery; and to Mr. Abernethy the credit is due of extending the practical application of the views of his master, as well as of promulgating much which was original, and, by his influence as a teacher of surgery, directing attention very generally to the importance of the investigation. Mr. Travers is one of the same school; not, indeed, a mere commentator or expounder of the opinions of others, but one who prefers (having the same end in view,) to choose for himself the road which appears to him most likely to lead to it.

* General System of Surgery.

Mr. Travers has devoted two volumes to Constitutional Irritation: the first appeared in 1827, and has reached a second edition, which, for a work requiring the exercise of considerable attention and reflection on the part of the reader, is a high testimony of the estimation it is held in. The second volume, with which we are now concerned, is introduced with a modesty worthy of the author's reputation.

"I request of my reader to bear in mind, that it professes to be only an 'Inquiry,' seeking and supplying illustration from facts, and reasonings thereupon; not aspiring to guide his views so much as to unfold my own, and thus to indicate the direction in which, as it appears to me, his also may be usefully applied." (*Pref.* p. viii.)

In the former volume Mr. Travers considered that disturbed state of the vital functions usually called constitutional irritation, which is wholly dependent on local injury or operation; he devotes the present volume to the effects of a morbid state of the constitution on local injuries: the first he calls "direct constitutional irritation," the second "reflected irritation."

But, before entering on the detail, it will render the subject more clear if we endeavour to give a sketch of Mr. Travers's pathological principles, and a general outline of the plan of the volume; premising, however, that in the present article we shall confine ourselves to the subject of reflected irritation, leaving the second part, which embraces the pathology of the nervous system, for future consideration.

Mr. Travers considers the nervous principle indispensably necessary to every vital action. Its disturbance constitutes irritation, and, according as it interferes with the properties and actions of other parts, the symptoms of such interference are manifested. As by sympathetic connexion all parts are liable to be consensually affected, local and constitutional irritations are reciprocated. Thus, sympathy may be said (by a metaphor,) to be the carrier, and irritation the thing conveyed. "If an irritation, strictly local in its origin, produce a certain degree of reaction, the determination to the nerves and blood-vessels of the part presents a series of phænomena constituting inflammation." This may begin and terminate in the part, and thus be strictly local, or the local action may be of such a character as to excite the sympathy of the system, and produce fever; and this constitutional irritation may serve a salutary purpose, and be indispensable to the recovery of the part.

"When the injury, from its nature or its seat, is one of extraordinary severity, the constitution is affected to great febrile excitement or depression, as acute inflammatory or typhoid fever, but still presenting the type of fever. In extreme cases, however, as those of complicated injury, although neither involving vital organs nor the loss of blood, it loses that character altogether, and the system is permanently sunk beyond recovery, or is stunned and prostrated for a time, and recovers only to display new and anomalous modes of excitement." (P. 43.)

This then is direct constitutional irritation, to which Mr. Travers has devoted his former volume. Reflected irritation (with which we are now concerned) "is that in which the system operates upon the part, or upon any remote part, prejudicially, or in a manner destructive to its own safety. This is exemplified in the untoward actions of erythema, erysipelas, gangrenous inflammation, supervening upon injuries; the insidious inflammation of remote parts, as the membranes of the brain, chest, or abdomen, or some visceral disorganization; and, lastly, in the preternatural actions of the nervous system, as trismus and tetanus." (P. 43.)

These two conditions are thus further explained and contrasted:

"Constitutional irritation I consider to be of two kinds, direct and reflected; by which arbitrary distinction I mean to imply, that the first is wholly and immediately derived from the part, commences and is identified with the local mischief, and the constitution has no share in its production. The second, on the contrary, originates in a peculiar morbid state of the constitution, to which the injury or inflammation has given birth, or it may be previously existing. The first is truly symptomatic; never originating spontaneously, and, being immediately induced by the local irritation, is capable of being essentially mitigated or arrested by its removal. The second is occasionally idiopathic, and, being as often the cause as the effect of the local action, is less influenced by local treatment. In the first, the local changes are depending on local causes; in the second, they depend on constitutional causes. Cases are of no uncommon occurrence in which, after an interval, the reflected supervenes upon the direct irritation, or the direct is superadded to the reflected, already established; which may therefore be regarded as examples of mixed irritation, the part and the constitution acting and reacting alternately upon each other." (P. 2.)

We cannot agree with Mr. Travers that the nervous principle is indispensably necessary to every vital action, because vital actions are sometimes carried on (as in the whole vegetable world) without the presence of any nervous system whatever. That irritation, however, is a disturbance of the nervous agency of a part we admit, as the symptoms fully prove it; and it is also most probable that the nerves are the channels through which sympathy takes place, The term irritation, although very commonly employed, yet is used with so little precision, and so mixed up with theory, as to convey different meanings by different authors: it is therefore of importance, in the first instance, for every writer clearly to state what he means by it. It enters largely into, and indeed is almost the foundation-stone on which is built, the physiological doctrine of Broussais, who considered it to be merely an exaltation of the vital action of the part, differing from healthy excitability on the one hand and from inflammation on the other, only in degree; being more than the first, and less than the second: to irritation, varying only in degree, he attributed every morbid change, so that all diseased actions were merely owing to an increase or decrease of healthy ones. Hence the term "physiological doctrine," as applied to the explanation of disease. If we understand Mr. Travers, he employs Irritation (in the true spirit of a Hunterian,) to denote an action which differs not in *degree*, but in *kind*, from any healthy action, or even from the diseased actions to which the term inflammation is applied; and therefore, in the strict sense of the term, a pathological phenomenon, and not a physiological one. In this view of the matter we fully coincide, as well as in Mr. Travers's objection to the attempt made by another celebrated French theorist, whose eclectic doctrines have superseded at present the "physiological" one, to do away with the term inflammation, because, from the vagueness of its application, it has produced error and confusion with reference to processes which are altogether different; and, instead of grouping under one head a certain set of changes as results of inflammation, to study in the dead body only the changes which are produced, and to classify them according to their physical characters. Believing, however, that John Hunter's pathological principles were correct, which accounted for all morbid changes by the perverted actions of the natural and ordinary powers of the parts, we assent to the introduction of the term irritation to express the disordered action of the nerves, as we do of inflammation to express one of the kinds of faulty actions of vessels; and agree with Mr. Travers in believing that irritation and inflammation are distinct actions, although the one often merges in the other. The term "reflected irritation" is a metaphorical one, which serves as a short definition of a phenomenon that would otherwise require the frequent repetition of a more lengthy paraphrase, and on this account (when its meaning is completely mastered,) is convenient. Of course, it throws no additional light on the action itself. Mr. Travers admits that "it is a monstrous evil in science to multiply even synonymous terms;" and, as he has evidently reflected much on the subject, we must give him credit (notwithstanding our dislike of such innovations,) for not having been thoughtlessly guilty of the fault he deprecates.

Mr. Travers enters at some length, particularly in a supplement to his previous chapters, into his own views of the nature of irritation, inflammation, and sympathy, of which we have given a faint outline, dwelling more particularly on the great principles. And let it not be thought that such reasonings are not of practical importance. The deleterious influence of erroneous generalizations, that is, of false principles, in medicine, can be duly appreciated alone by those who have studied the literature of their profession; although, unfortunately, the most superficial observation on what is going on daily around us proves that the same error is hourly committed by those who pride themselves the most on being purely practical, without being at all aware of their own theorizing propensities, or even by those who never think at all about the matter. What, indeed, can be a more convincing proof of the influence of theory on those who would have been supposed to be the least disposed to be led astray by a disposition to theorize, by all who regard the disposition as the peculiar fault of book-men, than the heating treatment of eruptive diseases. Brandy and water, cradles covered up with shawls and blankets, windows and doors hermetically sealed, are modes of putting into practice that theory to which the ignorant in general, and nurses and other old women in particular, still vigorously cling, of "throwing out the eruption." We will not say that the physiological doctrine of Broussais has had so destructive an influence on human life as this; for it has been embraced by those who, from their education, are more open to the conviction of experience than the less learned and more inveterate theorizers we have just alluded to; but that the application of a theory based on the assumption that all diseases are the result of different degrees of development of a physiological action, and therefore require the application alone of various degrees of the same plan of treatment, the antiphlogistic, will strike every experienced man to be fraught with danger, or at least likely to lead to very unsuccessful practice.* Such being the results of bad reasoning, it is by no means a useless task to test the correctness of any generalizations, whether begotten by the scientific or the ignorant.

The subjects which are treated at length by Mr. Travers, as examples of "reflected irritation," in this volume, are, 1, the unfavorable influence of previous organic disease on local injuries or operations; 2d, metastasis; 3d, secondary inflammations, either in parts adjoining or very remote from the local wound or injury; 4th, morbid growths independent of inflammation, and only arising in certain habits of body; and, 5th, inflammations, as erysipelas and gangrenous inflammation, often excited by wounds and injuries, but of which the origin and progress are strictly indicative of a faulty state of the constitution.

The consideration of these subjects is preceded by a few observations on the influence of constitution and habits in predisposing the body to disease. Mr. Travers believes that, "as there is no human condition free from trouble, so there is not an individual in existence who has an organization of equal perfection;" and that this fault of the constitution has been "both acquired and accumulated by the unceasing operation of deteriorating causes upon the human

* That it was so even in the hands of its gifted founder, is unfortunately shown by a table of the mortality at the Val de Grâce during several years, where the relative mortality in the wards of M. Broussais is compared with that of his more old-fashioned colleagues, which will be found in a work entitled "De l'Irritation et de la Phlegmasie, ou nouvelle Doctrine Médicale, par V. Prus, D.M. 1825." This work is curious, from its containing no reference even to John Hunter's name, although it was a Prize Essay on Inflammation, and more Hunterian in its principles than most of those emanating from the French schools.

body, from which our first parents were as far exempt as was compatible with the condition of perishableness by age and slow decay." The most influential predisponents to disease are "of the time of life, youth or age; of temperaments, the scrofulous; of habits of life, those of poverty in the aggregate; of moral causes, anxiety and the depressing passions; of external and adventitious circumstances, the abuse of liquor, the operation of cold, and the action of mercury. But it is in combination that these latter, which may be called occasional causes, act with most fatal severity." (P. 9.)

1. *Pre-existing Organic Disease.* Mr. Travers considers that a change from the normal structure, on account of the insidiousness of its origin, is often overlooked. He is inclined to think, however, that such changes of structure exist more frequently than is supposed.

"Upon examination of the bodies of persons dying from disease or accident after the middle of life, it is rare not to meet with some palpable evidences of organic change not previously known, or at best only vaguely surmised to exist. I could cite many instances of this, and also of chronic states of disease, explaining the adverse results of operations, contrary to the expectations of the surgeons. Diseases sometimes lie hid, as if they had by slow induction and encroachment inured the system to bear with them; or the symptoms have been masked, perhaps neutralized, by combination with others. I have known a stone in the bladder discovered after death in the body of a man otherwise diseased, in whom no complaint during life had led to a suspicion of its presence.

"On the other hand, the pain attending some very slight and superficial morbid change is sometimes excessive, and very difficult to comprehend.

"The extent to which the deterioration of organs, as life advances, is evinced in their interstitial deposits and general loss of transparency, sharpness, and firmness of texture, constitutes a sort of microscopic morbid anatomy, which for the most part escapes observation. It is most perceptible in the bodies of persons of middle age; the cases of premature decay, in which people are vulgarly said 'to die of a broken heart.' Anxiety and alcohol are the chief exciting causes, especially when, as too often happens, they co-operate. The effect is a preternatural flaccidity, or general loss of tone, with effusion from the extreme vessels. This slow and silent work of deterioration begins earlier in some constitutions than others, and varies in families as in individuals, notwithstanding wide differences in the habits and modes of life, as if from an original conformation; and it is thus we must account for the well-known and well-established fact, that, barring accidents, the general duration of life tallies in families, with few exceptions,—some reaching threescore years only, others five years over that, and others, again, reaching seventy, and from thence longevity." (P. 11.)

Mr. Travers justly remarks that we are often restrained from offering the chance of operations by the actual demonstration of internal disease. He refers to instances of fatal results having followed trivial operations, which can only be explained on the supposition of the existence of some visceral disease.

"A morbid condition of the lungs or liver, and effusion into the chest or upon the brain, have generally been indicated in such instances. In other cases, however, where examination has been obtained, no actual change has been detected beyond a gorged condition of the blood-vessels of these organs, partial depositions from their extremities, opacities, and old adhesions of the lining membranes, extreme flaccidity of the muscular fibre, &c." (P. 13.)

We believe that few practical surgeons will be inclined to dissent from the truth of these observations. That organic disease to a great extent may exist, and yet furnish no indication of its existence by symptoms during life, will be readily acknowledged by all who have directed their attention to necroscopic examinations. In the works of various pathologists instances of the same kind may be found; and Mr. Travers refers to a case of stone in the bladder, which was never suspected to exist during life. Andral, in the fifth volume of his "Clinique Médicale," mentions cases of softening of the brain, only detected at the post-mortem examination. These, and similar instances of every-day occurrence, should only lead us to prosecute with more industry our pathological and semeiological investigations. The fatality of relying on the appearance of symptoms alone must now be generally acknowledged, and the importance of combining them with physical signs, whenever these latter can be detected, must also be granted by every one who has attempted to arrive at a just diagnosis of disease. We have only to look back a few years, before the works of Laennec and other pathologists appeared, and contrast the state of knowledge at that period with respect to diseases of the internal cavities with what it is at present, and we shall be at once sensible of the advance of modern medicine in this particular. The investigation of the physical signs of disease may be considered to be yet in its infancy, and we may hope, from what has hitherto been done in this way, that the art of diagnosis may be able to reach a much higher state of perfection than could have been anticipated even by its most sanguine cultivators. The importance to the surgeon of being able to detect visceral disease previous to an operation, we shall presently have occasion to refer to.

Four cases are given by the author as confirmatory of his opinion that the fatal result of surgical operations is the consequence of existing changes of structure in some of the internal organs. In the first case, sloughing of the cellular membrane of the scrotum followed from puncturing a hydrocele, and death took place the twenty-fourth day after the operation. The fever which accompanied this local affection began on the eighth day after tapping the hydrocele. The patient had been in the habit of drinking porter daily to excess. The post-mortem appearances were—sloughing of the tunica vaginalis; the testes and cord were sound, with a small hydrocele of the latter. There was a gorged condition of all the viscera; the liver was brittle and granulated. Some chronic adhesions were found in the chest. In the second case, peritonitis followed by death took place from tapping an ovarian dropsy. On examination, a small fungus, of malignant aspect, was seen sprouting from the enlarged and hardened substance of the right ovary. Death followed the opening of a bursal abscess in the third case, the subject of which was a girl, aged eighteen, an epileptic. On examination, the affection was found to be confined to the bursa. The mucous membrane of the bowels was ecchymosed in several places, the right lobe of the cerebellum was morbidly soft, and about an ounce of blood was shed over the surface of the left hemisphere of the cerebrum, beneath the dura mater. In the last case, a large muscular man received three contused wounds in the scalp. He died on the ninth day from the accident; and, on examination, the wounds were found in a state of slough, with the surrounding integument thickened. There was no injury to the tendon, pericranium, or bone. The pia mater was very vascular, and the sinuses were turgid; the arachnoid was transparent, from a copious effusion beneath it. Old pleural adhesions were found in the chest, and the right lung was filled with tubercles.

Whether the organic changes detected in these examinations were sufficient to account for the untoward result of the operations must be left to the judgment of individual practitioners. In some of them we should be inclined to doubt it. Most surgeons of experience must have met with similar cases in practice, and it is very probable that, if diligent examinations were made, changes similar to those mentioned by Mr. Travers would be found. The deposition of tubercles in the lungs or intestinal canal is, in particular, too often overlooked by surgeons; and yet it can be easily shown that, if they exist to any extent, no rational hope can be entertained that a serious surgical operation will be attended with success. The removal of a local disease by a sudden sweep of the knife, so far from arresting, often materially contributes to hasten, their deposition. Minute ulcers of the gastro-intestinal mucous membrane may so depress the vital power as to render the constitution too irritable to accomplish the functions necessary for the restorative process after great operations: or some organic disease of the heart may exist, which will render it hazardous to amputate a limb, and thus compel the heart to circulate an additional quantity of blood.

2. *Metastasis.* Mr. Travers considers the principle of metastasis to be the same as sympathy, of which he gives a very curious proof.

"If a child dies at the seventh month; from the period of its death, though the woman be not delivered for days after, the mammæ are furnished in the same plenitude as if the child was born and alive. Now this is directly contrary to what writers on Midwifery have generally asserted, viz. that the falling of the breasts, and the failure of the milk, are never-failing signs of the child's death. This was misplacing the sympathy, the secretion being determined not, as this statement would lead us to suppose, by the life of the fœtus or the birth of the child, but vicarious with the diminished supply of blood to the womb on its death, or after delivery. Nature never contemplates death; and therefore, when the blood of the uterus ceases to be demanded, the child, in the language of sympathy, is born, and the want of the born infant is supplied." (P. 18.)

The most marked examples he has seen of metastasis are serous apoplexy, from retention of urine and the sudden reduction of swollen testes; palsy of the retina, from a sudden retrocession of a skin-rash, from a tumour obstructing the bowels, the irritation of worms, engorgement of the liver, and suppressed catamenia; and erythema, erysipelas, and even diffused gangrenous inflammation, from the healing of wounds upon distant parts.

With respect to metastasis, it has been observed that it frequently occurs in structures analogous in their anatomical elements to those primarily diseased; as in the case of pericarditis following inflammation of the fibro-serous membrane of the joints, &c. In three of Mr. Travers's cases, what he has supposed to be a metastasis arose from the healing of cutaneous ulcers or abscesses of the integuments, followed by affections of the mucous membrane either of the abdominal or thoracic cavity.

It seems to be a prevalent opinion that the older writers were disinclined to heal up old ulcers, from a fear that constitutional mischief should follow, rather on theoretical grounds based on the humoral pathology than from experience. Such being the case, the instances given by Mr. Travers are worth consideration. The history of some of these cases is too cursorily given, and so much difficulty hangs over the whole subject that they do not strike us as altogether conclusive instances. Some doubt, we think, must be felt by all as to the propriety of classing the following case under this head.

"A man who had a thecal and palmar abscess of the hand, following a puncture of the ring-finger, and was recovering a partial motion of the fingers, the tumefaction having subsided and the discharge nearly ceased, was attacked suddenly with acute inflammation of the chest, which resisted active treatment, and proved fatal in nine days." (P. 20.)

The following case appears to us much more conclusive.

"A gentleman, of dissipated habits, had a great parenchymatous enlargement of both testes, for which he applied a suspensory, and a strong solution of acetate of lead. The volume of the testes was suddenly reduced, and he was as suddenly attacked with a fit, resembling apoplexy: he never perfectly recovered, and eventually, though some years afterwards, died of a return of the stroke." (P. 22.)

3. *Diseased Actions set up in other Parts by Injuries and Inflammations.* The late Mr. Rose, of St. George's Hospital, was the first who particularly directed the attention of the profession to the frequency with which large collections of matter, consisting partly of whitish lymph and partly of pus, are found after death following a severe operation or injury. These abscesses are most apt to form when the parts in which the injury took place are in a state of suppuration, and in particular when, from the nature of these parts, after the confinement of the matter, great irritation of the system has been kept up; and it often happens, as Bertrandi remarked, that they have not been suspected during life. Desault had previously pointed out the frequency of abscess of the liver following injuries of the head. "Two possible sources of fallacy," says Mr. Travers, "are so obvious, that it is scarcely necessary to point them out. The first being Richerand's mode of explaining it, viz. the consentaneous injury of remote parts; secondly, the previous existence of disease. For the first, the physical impossibility of the occurrence in the majority of cases, and, for the second, the previous absence of symptoms, are sufficient to silence scepticism." We are not disposed to be over-sceptical, as there are too many cases on record of considerable deposits of matter in various internal organs succeeding operations, to allow of hesitation in admitting that the two are in some way connected as cause and effect; but we think that these explanations are of more weight than Mr. Travers allows, and in many cases may partially account for the phænomena. In the case of a fall on the head, for instance, we can scarcely say what injury the internal viscera may at the same time receive. We know, at all events, that the liver, in consequence of its friable structure, has been ruptured from injuries of the abdomen, the integuments remaining entire; a point which is much insisted on by Cruveilhier in his pathological investigations: and that Mr. Travers should rely on the absence of symptoms as being decisive of the non-existence of disease, appears to be at variance with his previous observations, in which he dwells particularly on the fact that organic disease may exist without symptoms, and gives in illustration an instructive case.

Granting, however, that the internal abscess is the effect of the operation, we have still to enquire on what the choice which was made of the peculiar organ for the purulent deposit depended. As it regards abscess of the liver following injuries of the head, Mr. Travers accounts for it by the well-ascertained sympathy between the two organs. Still, however, as it does not take place in all cases, nor in the same organs, we must further look to some local cause for the action in the viscus; and this may be either some organic peculiarity or some inherent disposition to disease, which in some cases may have been produced by simultaneous local injury. In other cases, organic disease previously existing, without external symptoms, may determine the situation of the secondary abscess.

As the investigations of pathologists since the time of Rose have increased the accuracy of our knowledge of these secondary purulent deposits, we regret that Mr. Travers takes no notice at all of them, except in a short passage at the latter end of this part of the volume, (p. 222,) in which he seems altogether to reject them, as destitute of credit. There may be much that is crude and hypothetical in the explanations in which some of these pathologists have indulged, but we may gather from their united observations that there are two distinct conditions of the parts surrounding the purulent deposits; one, in which there is complete absence of vascular congestion, lymph, induration, and softening of the surrounding tissues, the pus being collected in the natural texture of the part; and another, in which the tissues in contact are softened, or ulcerated, or covered with layers of lymph. Another fact, too, has been fully proved by them, that in nearly all these cases pus has been found in the veins of the part affected. There are therefore good grounds for the inference that pus may be absorbed by the veins, and carried to and deposited in a part previously (as far as appearances go) sound, or that the pus circulating with the blood may, together with other causes, induce suppurative inflammation in remote organs. From the same sources we learn that the lungs and the liver are most frequently the seat of these accidents, and the kidneys least so of all the internal organs; but, that Desault's opinion, that they are found in the liver more often after injuries of the head than of other parts, is not correct. This being the case, we can hardly assent to Mr. Travers's theory of the natural sympathy between various organs, directing the seat of the morbid process, which seemed a probable explanation of Desault's observation. The fact of the infrequency of the disease in the kidneys appears to militate against the influence of sympathy; for these organs are in intimate sympathy with the skin, a texture which is seriously involved in all operations. Although the first opinion expressed in the following quotation is questionable, yet the observations on chronic diseases supervening on injuries are highly judicious.

"The natural sympathy is equally remarkable, in most cases, between the brain and liver, whichever organ is first affected, and explains why this is the most frequent, and was the first-noticed instance; but the partial symptoms vary in individuals according to their temperament and habit; so that, in a person of phthisical diathesis, the weight of the injury would fall upon the lungs, whereas, in one subject to hepatic congestion, it would probably fall upon the organs included in the system of the vena portæ; but the variety of the casualties and the diffusedness of the mischief plainly show that, in the case of recent injury, it is by the medium of the universal sympathy that the site and extent of the morbid action are determined. But the result is not limited to recent acute injury; it is a chronic state of continual occurrence. Let any candid observer watch the progress of white swelling, of scrofulous swelling of the testis, of slow and extensive glandular swellings of the neck and groin, which suppurate deeply and imperfectly, of psoas and lumbar abscesses,—and, in short, of all such local diseases as have induced a chronic cachexia, and keep up an habitual hectic,—and he cannot fail to observe their tendency to produce visceral disease. If the case be one which admits of a palliative plan, and occurring in a child, he will probably see it sink into phthisis at puberty, or, by dint of incessant watchfulness, live a cripple and exotic. If the state of the part demands amputation, and over-anxiety to preserve it leads to a postponement of the operation beyond a certain time, the hectic continues, and the system succumbs before the wound is healed, instead of instantly rallying, as having cast aside its burden.

"The cavities or viscera of the chest or abdomen will invariably be found so diseased as to have precluded the possibility of recovery. Sometimes effusion on the brain with tubercles of the lungs; in other cases, purulent effusion in the chest or belly; mesenteric abscesses, or such patches in the liver or spleen as Mr. Rose has described; or complicated morbid changes produced by adhesions between contiguous surfaces and adventitious membranes, are found, forming insulated pouches or nests of matter, communicating by ulceration with the substance of the lungs, or some part of the alimentary tube, or the kidney and urinary bladder. * * * It would be as impossible to comprehend in a description the varieties of external disease leading to them as of internal changes produced. It is enough for my purpose to lay down as an axiom the fact, that the hectic produced and maintained by sympathy with an external disorganization long enduring, as, for example, a diseased knee or ankle-joint, becomes an exciting cause of various structural changes in the visceral organs, and offers the best apology for early, the strong objection to late, operations." (P. 29.)

These practical remarks are especially valuable when taken in connexion with the discoveries of M. Louis (to which Mr. Travers does not allude,) on the deposition of tubercular matter in internal organs. We refer particularly to the fact, so completely established by M. Louis, that tubercular formations, when detected in any of the internal viscera, are always found (except in young children) at the same time, and in a more advanced stage, in the lungs. Although the remarks of Mr. Travers are destitute of the numerical precision of the French pathologist, yet to a certain extent the former corroborates the latter; for, without admitting Mr. Travers's explanation that the external disease is the exciting cause of the internal, we have the fact itself that, at a certain period, the two affections are coincident; and, in a practical point of view, we shall endeavour to show that this coincidence is of importance. It is to be regretted that we are still deficient in more precise information on the surgical pathology of tubercular deposits. We have yet to learn the exact relations between the external deposition of tubercular matter in the glands of the neck and groin, in the ankle and knee joints, in psoas abscesses, &c., and the internal deposition in the lungs, mesenteric glands, &c. We cannot yet satisfactorily confute or confirm the vulgar opinion that the mere external deposit of strumous matter, as in the cervical glands, prevents the disease from "falling on the lungs." The extreme difficulty of detecting early tubercles in the viscera during life, and the infrequency of opportunities of examining bodies in which death takes place under such circumstances as to preclude the possibility of the fatal disease acting as an exciting cause of tubercles, render the solution of this problem very difficult. The examination of those cut off by accidental death whose glands were enlarged, or joints affected with strumous disease in an early stage, would, if collected in sufficient numbers, supply valuable matter; and that in process of time such data will be collected, we think may be expected from the attention which surgeons now pay to internal diseases: but, until we have such information, we hardly think that the truth of Mr. Travers's "axiom" is proved, although, as a general rule, the advice it contains is judicious, and would have been much more so, had Mr. Travers taken more extensive views of tubercular formations. And this is an instance of medicine and surgery being, in theory and in practice, one and indivisible. The discoveries of M. Louis prove that in the lungs is to be sought the maximum of internal tubercular disease in all cases; and Mr. Travers has shown the coincidence, in advanced stages, of the external tubercular deposition with the internal. In all cases therefore of external scrofulous diseases which demand operation, the state of the lungs should be most carefully examined by physical means; and the evidence afforded, when taken in connexion with the other symptoms, should guide the prognosis. Every surgeon must have observed two sets of cases: one, in which the removal of the scrofulous knee-joint in an emaciated subject has been followed by the speedy disappearance of every bad symptom, and rapid restoration to health; and the other, in which the operation has been followed by an aggravation of all the bad symptoms, and death. The physician explains the difference by the fact, that, in the one case, the general symptoms were the product of mere local disease; and, in the other, of local disease together with a similar internal scrofulous disease, most probably of the lungs; symptoms which it is almost impossible to unravel and fix the due value of, unless by the aid of minute examination of the internal organs, and particularly of the lungs, by the assistance of physical examination of the chest. In the former case, there may indeed be tubercles in the lungs, which announce themselves by no sign; but it may be presumed that, on the removal of local irritation, these will remain in that latent state which seems to be compatible with many years of life, although of delicate health, still of usefulness and enjoyment.

In illustration of inflammation set up in other parts after operations and injuries, Mr. Travers details several cases. These are divided into instances of contiguous and of remote inflammation. As instances of remote inflammatory action, he gives cases of, 1st, pericarditis from a suppurating wound of the scalp; 2d, peritonitis from amputation of a leg for a fungoid tumour; 3d, and

4th, phthisis after ulceration of the cartilages of the knee-joint, and after a severe injury of the ankle-joint; 5th, adhesions of the pleuræ, inflammation and ulcers in the stomach, with enlarged liver, after a penetrating wound in the heel by a nail, which was followed by caries of the os calcis.

In the third and fourth cases, where death took place from phthisis, we are not furnished with any particulars as to whether the chest was examined on the admission of the patients, or whether the individuals presented any local or constitutional symptoms of phthisis when they received the injury, or were attacked with disease of the joints. Some doubt also hangs over the last case, from the fact of ulcers in the stomach often existing without producing any marked symptoms.

These cases of organic disease supervening on local injuries, and producing death, are instances of what Mr. Travers terms direct superadded to reflected irritation; and under this head are to be classed nearly all the protracted cases of surgery. The local disease, by its constant irritation, produces a faulty state of constitution, and tubercular or other depositions in the lungs or other viscera is the consequence of this "reflected irritation," which depositions become in their turn causes of direct irritation.

"It will be obvious to reflection, that the direct irritation ensuing upon injury or inflammation tends, if continued, to the production of the state of constitution above described; as, when the injury, which acts as an exciting cause, though soothed, is unredressed, and the system is, by the aid of time and circumstance, restored to seeming tranquillity, though still oppressed by a sense of inequality to sustain the demands made upon it for support and repair. In this critical position, it continually happens that the constitution of the robust, which is unused to confinement, and of the weak, which is scarce adequate to maintain health, betrays a condition of morbid irritability which impedes the process of recovery, and lapses sooner or later into a wasting hectic, generally producing pulmonary or other visceral deposit. In those chronic changes of structure which arise independent of any assignable local cause, some of which we denominate constitutional diseases,—as, for example, scrofula, scirrhus, &c.,—a similar passive morbid state of the system is gradually established, which, if aroused by any suddenly altered condition of the part, frequently assumes a degree of activity destructive to life. The irritation of a change, abstractedly beneficial,—as, for example, the removal of a tumour, or of a limb which we have vainly attempted to save, or the discharge of a large abscess,—annihilates the benefit which, in different circumstances, it would have afforded. Had not the powers of the constitution been already overstretched, they would have rallied on the occasion, as they have often done from a state of almost hopeless depression; but the shock incidental to the change of condition oversets it, so exquisitely delicate has the balance become by gradual adjustment between the weight imposed and the sustaining force. If fatal results are exceptions to general experience, it is because experience has impressed caution; but they occur; and it has appeared to me that a more distinct solution of the question, why they occur, than has yet been given, may render them still less frequent.

"This, then, is the case of the direct superadded to the reflected irritation; so that the reciprocity of the two is evinced in almost all protracted cases of surgery, and indeed their protraction is generally depending upon it. In a case which goes healthily forward without interruption, the constitution and the part preserve their adjustment so equally, that irritation can scarcely be said to exist; and this is the case when, after the first alarm and resistance of the nervous system is past, the circulation quiets down and lends all aid to the work of reparation. But, when permanently unfavorable circumstances attend an injury or a disease, an ill-disposed habit or a cachexia is present, a previous organic change exists, or an interference takes place with the operations of nature, whether accidental, unavoidable, or ill-judged, (each of which causes comprehends a variety of conditions in detail,) the part and the constitution are alternate irritants, one of the other, and eventually co-operate for destruction." (P. 44.)

From the cases given to illustrate the superaddition of the direct to the already established reflected irritation, we select the following:

"A young lady, a native of the West Indies, receiving her education in this country, became my patient some years ago for a painful affection of the knee-joint. She had been for some time confined to her sofa, and had tried liniments, local bloodletting, and the various applications in ordinary use.

"Finding the pain obstinate, I opened a large blister, fairly removing the cuticle over the joint, excepting only the part covering the patella. The suppurative inflammation was readily established, and the secretion abundant. The constitution very soon showed signs of great weakness and distress; nausea and fever followed. She complained of excessive soreness of the part; the raw surface began to ulcerate deeply, and the granulations turned sloughy. Mild cerates and poultices, which had been substituted from the time at which the sore became inflamed, the solution of opium, and other anodyne fomentations, were of no avail to arrest the sloughing process, which completely exposed the ligaments of the joint. The discharge ceased, and the surface presented an appearance resembling dry gangrene. Opium, ammonia, sarsaparilla, bark, and the other remedies adapted to sooth and support the system, with a careful administration of cordials, were employed to no effect: a state of extreme prostration and irritability ensued; the integument of the sacrum took on the same condition; she became typhoid, and died about three months after the application of the blister.

"The West Indian constitution is one of feeble powers: though from her infancy inured to this climate, this young lady was of slender frame and strumous diathesis; she had suffered in health from the confinement, being incapable of standing and of moving the joint without pain. The destructive process was similar to what is more frequently seen in weakly children. It is the only case in which I have seen a blistered surface fall into gangrenous phagedena in the adult." (P. 53.)

The next division of Mr. Travers's work embraces the consideration of local changes of structure not essentially inflammatory, and of the peculiar states of constitution, or cachexies, on which they depend; on which account they come under the head of "Reflected Irritation."

Mr. Travers believes that we are too much accustomed to consider every change of texture, or deposition, a result of inflammation; and that, to account for the different products, we make out not only acute, sub-acute, and chronic, but scrofulous, scirrhous, rheumatic, gouty, and venereal inflammations; to each of which a peculiar action is assigned. But

—"inflammation is in itself the same, although its character and products vary according to the influence exercised by the constitution, and the term specific is used to designate such actions as are characteristic of a certain previously existing state of constitution, and peculiar to it. Admitting this, I contend that many effusions, depositions, and absorptions, both simple and specific, are in no degree inflammatory." (P. 66.)

The misconception has principally arisen from the circumstance of inflammation being superadded to such changes in the progress of disease.

"When a lymphatic gland, instead of its proper pulpy texture of a greyish brown colour, is found to consist of a firm suety substance, or to contain a white curd-like fluid; or when the proper texture of the testis is so converted, with a very moderate or no increase of bulk, and preserving its uniformity of figure; when there is neither pain, heat, nor redness, nor any sign of increased vascularity, nor any discoverable derangement of health, what reason have we for considering it as inflammatory action?"

"How are we to reconcile the phænomena of many dropsies, as of the cellular membrane in simple œdema and anasarca, of congestive ascites, of transparent hydrocele, hydrops articuli, of adipose and fatty tumours, and steatoms, and such sebaceous cysts as those common to the face and hairy scalp, with the idea of inflammatory action?

"Inflammation doubtless may give origin to these and similar changes, as it produces an opacity of the crystalline lens, although the cataract in the ordinary form of the disease is wholly unconnected with an inflammatory process; and this in proportion to the former species is more frequent.

"The elementary structures of scrofula and cancer are themselves produced independent of inflammatory action, and are strictly speaking incapable of the production of adhesive lymph or pus; it is only when inflammation is superadded to them that secretions at all resembling these are formed." (P. 68.)

If a cyst containing a glairy fluid, which has been attended previously with no inflammatory symptoms, is opened, or becomes inflamed by distention, its secretion then becomes mixed with the true products of inflammation.

Neither morbid secretions nor slow alterations of structure, improperly termed "chronic inflammations," if unattended with the acknowledged evidences of inflammation, can be properly said to arise from it. Indolent tumours, in general, whether solid or fluid, give no such sign.

"The origin, there can be no doubt, of these swellings and collections is due to a distinct series of actions, the obstruction or deficient action of absorbents, or the preternatural out-pouring of the excretory and exhalent capillaries from congestion of the venous circulation and other causes." (P. 70.)

The sections of all varieties of solid tumours, the adipose, sarcomatous, mammary, osteo and fibro-sarcomatous, warts, condylomata, tubercle, medullary, hematoid and melanoid fungi, present such varieties, and such an entire absence of every symptom and ordinary product of inflammation, that they can only be regarded as the results of a morbid irritation, whether with excessive or defective action of the capillaries of the part. In a large proportion of the cases of changes in the solids of the body inflammation is not an antecedent but a consequent, and either excited by distention or the falling together of parts from loss of substance and support. The antecedent is local irritation, the consequences of which, when the system is in health, are "for the most part simple and unimportant. If inflammation supervenes, it runs its course, and leaves the part unimpaired. But it is not so if the system is either not in present health or not a healthy one by temperament or circumstances, as in the scrofulous or cancerous diathesis. And thus the very trifles which are utterly disregarded by some persons, in others form the fuel, or kindle the flame, of incurable maladies." (P. 75.)

"The symptoms which attend upon these chronic changes are a sensation of general malaise, languor, and lassitude, wandering pains, unequal distribution of blood and heat, uncertain rest and appetite, gradual decline of muscular tone and power, and perhaps of flesh, variable secretions both as to quantity and kind, and an uncertain state of the temper and spirits. These are the signs of irritation from chronic change, and are indicative of the state of the system which has given origin to the morbid action as much as of its effects. I call it, therefore, reflected irritation.

"The cases of extensive chronic change, which, unconnected with a specific diathesis, endure for years, causing great inconvenience and great deformity, without producing one constitutional symptom of a truly inflammatory character, form a very large class of surgical diseases. But, when the state of inflammation supervenes, and leads on to the institution of the hectic paroxysm, thenceforward the wear and tear becomes destructive; and, unless the cause admits of speedy removal, it is not the external malady to which our attention and our fears are directed, so much as to the organs which support life, especially the lungs.

"The irritation of the constitution is barely perceptible in the early stage of the specific diseases. In scrofula, in all the varieties of cancer, in the numberless tumours and ulcers which have no obvious and direct local cause of origin, the constitution which has given birth to them being predisposed to their formation, discovers little sympathy with them when formed. The seeming exceptions to this observation are those set up by violence, or whose situation interferes with the structure and function of vital organs,—as tubercles in the lungs, brain, stomach, or liver, for example.

"Then the irritation becomes direct, and sets up fever, which rapidly proves destructive; for reflected irritation, if left to run its course, is slow in comparison with the direct, which, though originating in some purely local cause, acts, as we have seen, with such intensity as to excite the instant sympathy of the brain and stomach, and thence of the universal nervous system." (P. 75.)

Mr. Travers further illustrates the proposition of inflammation supervening in constitutional diseases by a reference to the phænomena of specific diseases. Thus, the tumors of scrofula, and the scirrhous tubercle, are not generally painful until they take on inflammation. The venereal poison is introduced into the system by means of an inflammatory process, often very trifling, and it is by the inflammation of distant parts, (as eruptions, sore-throat, ophthalmia,) modified by the poison, that its presence in most cases is demonstrated. This, then, is a proof "of a morbid state previously existing, an irritation reflected from the system upon the part, and shewing itself in the specific type of its inflammatory changes."

Mr. Travers believes that a morbid poison received into the circulation will sooner or later show itself in an external form; but, independently of that, it will give notice of its existence by a visible decline in the functions of health. Nevertheless, he conceives that "a man may die of scrofulous or venereal irritation and atrophy, without an external or local sign demonstrating its interference with the vital economy."

"I consider inflammation specific, in being subordinate to the specific irritation; and, when this is the case, it is of a chronic character, and circumscribed, as in the scrofulous white swelling, in the scirrhous tumour of the mamma, in a genuine syphilitic ulcer of the glans penis. If the action is rapid and vehement, and threatening extensive disorganization, we may be sure it is the work of some other agent in combination, as, to take the last example, a state of high inflammation from neglect, or venereal abuse during the existence of sores; confinement of matter, as in perfect phymosis; intemperance, or the previous employment of mercury in frequent irregular courses during unrestrained exposure to weather. The same remark applies to venereal ulcers of the throat and external skin, to eruptions, nodes, venereal ophthalmia: take away the susceptibility to inflammation from exposure and other incidental causes during the excitement of the system by mercury, and the severe local consequences referred to the action of syphilis would be no more seen." (P. 80.)

The above remarks are highly important, and should be well considered by every practitioner. The complications every day met with in the treatment of syphilis, we believe, in a great measure will be found to depend on inflammations supervening on the original affection. That, in the syphilitic subject, as Mr. Travers states, irregular living, a blast of damp air, or chilling the surface, will give rise to an inflamed iris or ulcerated throat, or nodes, with blotches on the skin, we are not exactly prepared to affirm; but, as a general position, we agree with Mr. Travers in thinking that injudicious treatment, intemperance, cold, &c., are the real causes of the various complication of the diseases above alluded to. We are aware that the term specific inflammation is objected to by many, particularly by the disciples of the French school; but, as the author observes, the liability of particular textures to be affected with particular inflammations affords a strong ground for the propriety of dividing inflammation into specific and simple; particularly when some of these inflammations are found to be more within the influence of certain remedial agents than others.

Mr. Travers states that there is another view in which the specific diseases deserve to be regarded. In this view a constitutional disease may have indirectly a local origin, and that one of simple inflammation. He takes scrofula as an example, and observes that "the habit most alien from it may be rendered truly scrofulous in three months, by a combination of such causes as will produce that state with a local casualty. An impoverishing system of diet and medical treatment, a vitiated atmosphere, with bad nursing and neglect, or mistreatment of the local malady, will, and often does, effect this among the poor."

"Thus, it continually happens that the labourer works upon a strain or a wound, which, with rest and attention, might have been permanently cured, until, by extensive local disorganization and loss of health, he is forced to seek parochial or hospital relief. With great attention, the local disease assumes a favorable condition, and is approaching a cure, when the increased emaciation, sloughs of the sacrum, or trochanters, the small cough and flushed cheek, uncountable pulse, frequent diarrhœa, and profuse sweats, show that his lungs are irrecoverably diseased; and thus the whole career, from full health to the grave, is run within a twelvemonth." (P. 83.)

Mr. Travers next considers the state of the constitution resulting from the operation of specific diseases, "which has been well denominated cachexia, and which is, in fact, the peculiar action engendered by the specific disease, retaining the distinctive character which that possessed."

The usual acceptation, we believe, of the term cachexia, has been that in which Dr. Clark applies "tubercular cachexia," to denote certain symptoms marking a general faulty state of the whole constitution, which is the remote cause of the deposition of tubercle, and which results from the operation of certain external or internal agents, as a close atmosphere, damp, bad food, &c.

In mercurial and syphilitic cachexia, the general faulty state of the constitution is produced by the circulation of a morbid poison. Mr. Travers must therefore use the term "specific diseases" in a sense peculiar to himself, or he must understand by cachexia that condition resulting from the actual development of the disease, (as from softened tubercles,) and not preceding it. If the latter is his meaning, we must dissent from it; as the general condition on which tubercles or carcinoma, for instance, depend, appears to differ only in degree from the state of the body after these diseases are developed; that is, it is less marked. To us the definition appears obscure.

Mr. Travers thus classes Cachexiæ:

"SIMPLE CACHEXIÆ.
Suppuration.
Ulceration.
Gangrene.

SPECIFIC CACHEXIÆ.
Scrofula.
{ Scirrhous Cancer.
{ Lupus. Sweep's Cancer.
{ Medullary Cancer.
{ Hæmatoid. Melanoid.

Animal Poisons.

{ Syphiloid.

{ Varioloid.

{ &c. &c.

Mineral Poisons.

{ Mercurial.

{ Arsenical.

{ &c. &c." (P. 90.)

Suppuration taking place in the nates, around the anus, in the groin, axillæ, &c. in broken-down constitutions, is a form of cachexia, and the greatest benefit is obtained by well-applied rollers.

Ulcerations, particularly of the extremities, is another example of cachexia, and in hospitals the effects of rest, pure air, and good diet, strikingly show their dependence on constitutional causes.

Gangrene, sometimes following the slightest accident, and independent of a diseased state of the heart and blood-vessels, is also a sign of cachexia from depressing causes.

Of the specific cachexiæ, the scrofulous is well known. The medullary cancer, according to Mr. Travers's views, which appear highly probable, is the cancer of scrofula.

"The constitutional changes which more particularly characterize the syphilitic cachexia are, the reduced flesh and tone, the loss of complexion, activity, and vigour; the frequent rheumatism in various parts of the body, especially the head and chest; sensibility to cold and damp weather, and small febrile paroxysms in consequence of exposure. The digestive organs are very feeble, and the breath and perspiration have a tainted odour. Mercury, it may be said, has much to do with the production of this cachexia: as an aggravant, I do not deny this to be frequently the case, but not as an element.

"The mercurial cachexia is characterized by irritable circulation, extreme pallor and emaciation, an acute and rapid hectic, and an almost invariable termination in phthisis." (P. 87.)

The whole of this chapter, as well as the numerous cases which illustrate it, will be found to be full of instruction and interest.

An example of mercurial cachexia is related, of which the subject, a respectable married woman, had been for ten months a patient in a country dispensary. Her mouth and nose had been in a diseased state for a period of fifteen months. She invariably declared that she never had the venereal disease in any form, but had been incessantly taking mercury. A round excavated ulcer existed in the middle of the soft palate, and another of a similar description on the inner surface of her upper lip. Several small portions of bone had come away whilst she was under the influence of mercury. She was at first ordered two grains of quinine three times a day, with a lotion of nitrate of silver. This treatment was continued for three weeks. The ulceration somewhat extended, and she suffered from pain in the ulcers and headach. The blue-pill combined with opium was given, and porter was allowed, and for a short time with seeming benefit; but in about a fortnight it was evident that the disease was assuming a malignant character, and the medicines were discontinued. For five or six weeks more, the ulceration continued to extend, and the patient's health gradually declined. It was then determined to try the effect of the alterative and tonic action of mercury in this form: —℞. Hydr. Oxym. gr. iij.; Micar. panis, ʒj.; p. Conii, ʒj.; Aq. dist. q. s. M. et div. in pil. xxx. e quib. sum. j. ter quotidie.—Extr. Sarsap. ℥ss.; Dec. ejusdem, ℥xvi. M. cap. part. $3^{m}$ c. sing. pilula. She was at the same time ordered to leave off every application in the form of lotions, and apply dry lint to the ulcerated surface on the lip, and over that to make gentle pressure with strips of adhesive plaster, just sufficient to support the edges. The effect of this trial was decidedly favorable, and in about a month she left the hospital well in health; the lip, as might be expected, not quite possessing its natural appearance.

Mr. Travers considers this one of many cases in which mercury had been largely used, under a mistaken idea that the affection was venereal; whereas, it was originally a simple inflammation of the membrane lining the roof of the mouth, exasperated to destructive ulceration by the abuse of mercury. The case is further interesting as showing that the disease only eventually yielded to the tonic influence of mercury and sarsaparilla; which, however, proves nothing as to its syphilitic character.

Such cases as the above are by no means uncommon. Without reference to this particular case, instances of what Mr. Travers would call mercurial cachexia, where the medicine has been administered for other diseases besides syphilis, frequently present themselves. Indeed, the remark may be extended to the injudicious and long-continued use of other active remedies which produce a cachectic habit of body,—such, for instance, as arsenic, iodine, &c.,—probably from inducing serious lesions in the gastro-intestinal tube. In cases of what Mr. Travers calls mercurial cachexia, considerable difficulty arises from the impossibility of ascertaining whether an original syphilitic sore existed or not. The causes of this obscurity are numerous and evident. From the position in which a sore occurs, it may escape the notice of the patient; nor can we always rely implicitly on the statements of the patients themselves. The propriety of withholding mercury when symptoms appear similar to those which, according to our author, characterize mercurial cachexia, cannot be questioned; although we are in many instances induced, in the latter stages, to exhibit it as an alterative, when, as in the case just alluded to, it often proves highly beneficial, particularly when given in conjunction with sarsaparilla and tonics.

Mr. Travers pursues his illustrations of reflected irritation by considering at some length Erysipelas and Gangrenous Inflammation. He observes, that he has shown that diseases of the constitution, taking on a local form, are not always of an inflammatory nature; but, when this local form exhibits inflammation, it is subjected to the influence of the constitution, and must be regarded as a constitutional inflammation. Strumous ophthalmia and strumous sore-throat are familiar examples:

"When we view," says he, "such an inflammation springing up spontaneously, as an action for which we can assign no external or visible cause, we see, in its simplest and most genuine character, the constitution developed in the part, which, for want of a better term, I have called 'reflected irritation.'" (P. 117.)

Mr. Travers's object is now to consider such constitutional inflammations following local injuries.

"Not that it can be doubted that in reality all such affections begin alike, and that it is the variety of occasional causes, internal and external, which operate to call them forth, and of the constitutions upon which they fall, and not that of the diseases themselves, which leads us so to distinguish them; the state of the habit being much like that of a mine charged with inflammable matter, which only requires a spark for its explosion. And two circumstances especially tend to corroborate this fact of their identical origin,—viz. the greater proportion of cases of grave injuries which display no tendency to these affections, and the very slight injuries which often call them forth. Thus, a slight abrasion or contused wound of the skin is a more frequent occasion of erysipelas than a compound fracture; and a puncture or superficial laceration of muscular structure a more frequent cause of trismus than the more extensive mutilation of a limb." (P. 119.)

Of all the diseases of the inflammatory class which come under this definition, Mr. Travers selects erysipelas, as that of greatest intricacy and importance. We shall confine ourselves chiefly to those points which refer more directly to the professed object of the volume, necessarily omitting that descriptive matter which is essential to the completeness of the treatise, but which is not peculiarly novel, although sound, judicious, and worthy of perusal.

The parts disposed to erysipelas are, 1st, the skin; 2d, the skin and subcutaneous cellular membrane; 3d, the mucous and serous membranes.

1. *Erysipelas of the Skin.* The erratic form is the more dangerous, and spreading by the part is less threatening in its character and consequences than spreading by the constitution, (that is, in distinct patches with sound interspaces,) as the latter is owing to a greater degree of constitutional sympathy.

"Like gout, erysipelas is dangerous in proportion to its diffusion, by this denoting the universality of that state of system which gives origin to it; and its appearing in distinct parts not only shows how completely it is constitutional, but how much the constitution is oppressed and unable to relieve itself.

"The tendency to appear at the same time, or in quick succession, in parts remote from the point of injury and from the stomach, is a character of that erythematous erysipelas which arises from the operation of animal poisons, whether admitted by the skin or the stomach, in certain deranged states of the nervous system following slight wounds: in such cases I have known the patches to turn gangrenous in a few hours after their appearance." (P. 122.)

2. *Erysipelas of the Skin and Cellular Membrane.* The œdematous is the most common form; the œdema being a mode of relief to the overcharged vessels. A second termination is by suppuration and sloughing of the cellular tissue; and a third by gangrene, in which the skin dies along with the cellular tissue. This is rare, and happens abruptly, and from purely constitutional causes; unless occasionally, but very seldom, from excessive distention, as in enormous fascial abscess, or disorganization from violence, as in bad compound fracture.

"Gangrenous erysipelas is distinguished from gangrenous inflammation by the prior existence of the erysipelas, and its confinement to the integument, though diffused over a large extent of surface, as the entire or the half of a limb, for example. In gangrenous inflammation there is no such diffused swelling and redness as belongs to erysipelas, prior to the appearance of the phlyctenæ or sphacelated spots. When actual death has taken place in erysipelas of the trunk or limbs, the signs of distinction become faint, and would vanish altogether, but that the extent and circumstances of origin of gangrenous erysipelas seldom permit of the maintenance of life long enough to observe the phænomena peculiar to it." (P. 124.)

Mr. Travers next adverts to the fact which was first insisted on by John Hunter, that, in these terminations of erysipelas, the adhesive process is passed over altogether. The absence of this boundary, which, "like the boards erected round a building under repair, precludes interruption to and from its neighbourhood, constituting the strongest local character of erysipelas." "Suppuration, when not so circumvallated, is an action without an object, and as purely destructive as gangrene." Even in the variety called "phlegmonoid," from the effusion being firmer, and conveying no sensation of fluidity, as in the œdematous and suppurative, it is not organizable lymph which is poured out, as in phlegmon, but the cellular tissue is distended with concrete albuminous matter, such as is seen after opening the vesicle of a powerful blister, and with this, in an advanced stage, pus and sloughs are intermixed. A similar effusion is found plugging the cellular tissue above a deep-seated tumour, and above a diseased joint, giving the preternatural depth and resistance to the incision in amputation. "It appears to retard suppuration, or by its bulk and solidity to restrict it within narrow limits." Mr. Travers has never seen it except in the extremities, and considers it absolutely insusceptible of organization. This variety frequently follows injuries of the hand, sometimes of the foot, and, if unrelieved, terminates in gangrene of the whole limb; whereas, in suppurative erysipelas, the skin is preserved at the expense of the subjacent texture.

3. *Erysipelas of the Reflected Membranes.* The conjunctiva, membrane of the fauces, air-tube, and rectum, and, Mr. Travers believes, of the pulmonary and gastro-intestinal surfaces throughout, are occasionally the seat of erysipelatous inflammation. It is rare, and chiefly in the immediate vicinity of the skin: it has an œdematous character.

"The excessive chemosis of the muco-serous membrane of the eye, which ushers in the acute suppurative ophthalmia, indicates, not less than its disposition to gangrene upon the cornea, that it belongs to this species of inflammation. In the pharynx and membrane of the glottis, it arises from cold and causes operating constitutionally, in which it sometimes turns gangrenous, as in scarlatina maligna; and from wounds of the throat, or caustic substances applied or swallowed, the obstruction to respiration from excessive swelling is sometimes such as to demand tracheotomy to prevent suffocation. * * * The contagious property belonging to erysipelas is strikingly manifested in some of these cases. I have known it fatal to three of the same family within the space of ten days. In the rectum, ligatures of internal piles and of bleeding vessels, necessarily including a portion of the submucous membrane, give origin to this inflammation; and I have seen it in several cases produced by the division of a fistula, in which erysipelas of the nates, and gangrene of the integuments surrounding the anus terminating the erysipelas, terminated also the patient's life. The peritoneum is exposed to this species of inflammation after child-birth, and I believe that a large proportion of the puerperal cases are cases ot erysipelas. The diffusion of the inflammation over the cavity, the non-production of adhesions, and the presence of flakes of albuminous matter floating in a curdlike serum, the sudden prostration of power, and the strongly-marked property of communication, whether by contact or atmosphere, lead me to consider it as of this description." (P. 127.)

There seems to be little doubt that puerperal women are subject to two kinds of peritonitis, one of a simple inflammatory kind, and the other which, from the reasons Mr. Travers has given, may be called erysipelatous; and this view of the matter will furnish some explanation of the various and opposite modes of treatment which, at different times and by different practitioners, have been practised and recommended. But this erysipelatous form of peritonitis is not confined to the puerperal state. Dr. Abercrombie has given some highly instructive cases proving this; and three or four cases have fallen under our own notice of adult females in the same ward being all attacked within forty-eight hours with peritonitis, which, from the early prostration of strength, rapidly fatal course, not yielding in any way to the usual treatment, and the peculiar flaky, curd-like effusion, without adhesions, were evidently of the same character.

Mr. Travers next considers the "obscure pathology" of erysipelas. He considers erysipelas to be a nervous inflammation, or an inflammation continued under the direct superintendence of the nervous system; and the following is the outline of his argument. The capillary vessels of the skin are the seat of the peculiar inflammation, and these are at the same time independent of the influence of the heart, as is proved by the phænomena of the circulation; and in intimate relation with the brain, with which the skin is directly connected by the nerves, whose sentient extremities ramify upon it. The phænomena of blushing, and the opposite state, prove the more direct sympathy of the skin with the brain than with the heart: the blush precedes the heart's action, if any be perceptible, as pallor precedes syncope. The inflammation of erysipelas precedes the fever, and such an interval is not peculiar to erysipelas, but occurs in cutaneous inflammation from burns and scalds, as well as after injuries of cutting, tearing, and bruising the surface, and tends to show that the sympathy in disease also is slowly evinced between the heart and the skin, as compared with that existing between the latter and the nervous centre. In the more vigorous inflammation, as the adhesive and suppurative, the heart quickly enough participates, as in symptomatic inflammatory fever. The causes of idiopathic erysipelas, Mr. Travers thinks, strengthen his argument. He enumerates sudden changes of temperature, humid soil and atmosphere, polluted air, indigestible and luxurious food, exciting or depressing passions, excessive fatigue, &c., and asks, are they not alike depressing? Is it not upon the brain and nervous system that they act directly, and with especial force?

"In both sexes, the young, fair-skinned, and delicate, are liable to traumatic erysipelas, when debauched in their habits and mode of life. The frequent substitution of ardent spirits for food among the lower classes, is a powerful predisponent to the inflammation of erysipelas and gangrene, when they become the subjects of local injury. The gastric erysipelas, which Desault seems to have erroneously regarded as the universal form, is met with in persons of sedentary lives, and what is called atrabilious temperament, and among hard drinkers. It is common to find erysipelas, like jaundice and dropsy, in company with diseased changes of structure of the liver." (P. 142.)

The symptoms, as well as the causes, are those of deficient power; as pain, general sense of weight, confusion, lethargy, deafness, and prostration resembling typhus even, although the erysipelas is confined to the extremities.

As Mr. Travers's argument in favour of erysipelas being a nervous inflammation extends over a dozen pages, it would be impossible to do it justice by mere extracts; and, if we have failed in conveying a clear impression in our own words, our excuse must partly be, Mr. Travers's want of logical arrangement and frequent repetitions. We should be very unwilling to assert that erysipelas is not a nervous inflammation, in the sense which Mr. Travers applies it; for we are disposed to think, with Mr. Abernethy, that, as a general rule, the faulty actions of vessels are the consequence of the faulty actions of nerves. But we do not think Mr. Travers's arguments, furnished by the natural structure of the skin and its healthy sympathies, at all prove that erysipelas derives "its local peculiarities from its seat;" as we find the same texture extensively involved, as in inveterate psoriasis and chronic eczema, without any peculiar disturbance of the nervous system. The independence of the capillary vessels on the heart is a condition of that system, not peculiar to the skin, and consequently proves nothing. It is probable that the nerves are involved in that general faulty state, probably both of fluids and solids, which a bad state of the constitution implies, and that morbid sympathies are the consequence; but whether the peculiarity of erysipelas is to be attributed in so great a degree to the nerves, we think is still a matter of doubt. The causes enumerated certainly depress the nervous system, but they also depress and impair every vital function, and are common to numerous and different diseases.

As it regards treatment, the general opinion has been that it is a disease of debility. The general fear of full antiphlogistic measures has doubtless been inculcated by experience of their ineligibility and danger.

"The truth is, that an hypercatharsis of very short duration, or the loss of a few ounces of blood, has often proved fatal in an erysipelas, and the disease is not unfrequently terminated on a sudden, or the patient narrowly saved from sinking by such volatiles and cordials as could be got down. At this moment there are hospital physicians, and others of equal repute, who treat the disease with ammonia, camphor, bark, and even alcohol, at its outset; others who treat it upon a purely antiphlogistic plan, but of these few venture upon bleeding, or more than a single and early bleeding, and they have speedy recourse to alimentary support to preserve the patient's powers. The truth, probably, in this, as in most instances, lies in medio; for it is a curious fact, that the success of the two parties is as nearly as possible balanced, so that the principle of treatment approximates more nearly than the details would lead us to suppose. This must arise from such modification in obedience to the same general principle as the circumstances of individual cases dictate; an explanation equally creditable to the discernment of the physician and his freedom from prejudice. A proof, too, that the disease presents great variety, or rather, perhaps, the constitutions in which it appears; and that the empirical (not to speak it profanely) is in truth the rational practice." (P. 145.)

Mr. Travers has found, with the exception of calomel and tartar emetic, in the onset of the disease, especially when attended with gastric irritation, the greatest benefit derived from the early administration of ammonia, and the different forms of bark now in use. He does not consider any external applications, beyond a cool and gently astringent lotion, or a light and equally consistent poultice, of any advantage. He bears his testimony to the value of incisions.

"In inflamed œdema, œdematous erysipelas, diffused inflammation of the skin from whatever cause, provided it be attended with swelling and tension,—whether soft and pitting, or firm and brawny; without any reference to the effused fluid, whether pus or serum, whether a circumscribed infiltration, or a diffused and heterogeneous collection,—a free and penetrating division of the integument is ascertained to be the most effectual mode of relieving constitutional irritation and saving texture. The relief is equally marked, even although no pus follows the incision, from the removal of stricture, and the escape of blood from the divided veins.

"Much mutilation and many lives have already been spared by this admirable practice. The incisions may be proportioned to the mischief to the extent of three inches, but seldom need or should exceed that space. They should penetrate the entire cellular structure; and, if arteries of a size requiring more than the pressure of the finger for two minutes be divided, they should be secured by ligature. The venous bleeding is beneficial; arterial is neither advantageous nor safe, as has been sufficiently proved.* Two, three, or more such incisions may be made, if an entire limb is affected. Of the puncturing practice I have no favorable opinion: it irritates, without effectually unbinding or unloading, and is a cruel prolongation of suffering, especially when the disease is situated on the face and head." (P. 147.)

* "If such patients are left but for a few minutes to bleed, they are left to die, and the object and end of this invaluable practice are misunderstood. A needless extent of incision not only increases this danger, but is open to other obvious objections."

These views are illustrated by cases, all worth perusing with attention.

Mr. Travers next considers Gangrenous Inflammation, as a second instance of a constitutional inflammation exemplifying reflected irritation. He commences by distinguishing gangrenous inflammation from gangrene. Gangrenous inflammation is inflammation of which the termination or crisis is gangrene: it does not therefore apply to those instances of gangrene which depend upon strangulation or arrested circulation from a change in the structure of parts; nor to instances of decomposition from heat, cold, or chemical agents. In such cases, inflammation is a consequence of the gangrene, not a cause, and often is a conservative process set up to circumscribe and throw off the gangrene. In these cases, the gangrenous part is dry, shrunk, and mummied; and, if a conservative process is set up, "the line of demarcation is announced by the deposition of adhesive matter; then the ulcerative action, beginning upon several points and proceeding along this line, gradually accomplishes the separation, while, at the same time, the construction of granulations out of the adhesive matter, constituting the third process, advances the final stage of repair, viz. the fabrication of the new surface. In the mean time the health is very little interrupted, the system being able to take and apply the support which the case calls for." (P. 164.) But, in other cases, the gangrenous inflammation may ensue upon gangrene, which is announced by swelling, livor, and acutely agonizing pains above the dry dead part; a common affection in aged, exhausted, and diseased persons.

"The contrasted appearances show the difference of the two affections, viz. the gangrene (of the foot or hand, we shall suppose) dry, cold, pallid, shrunk, and insensible, and the gangrenous inflammation of the leg or arm, swollen, moist, livid, vesicated, acutely painful, and undefined in extent. In this case, the adhesive inflammation fails, and the gangrenous inflammation succeeds, and our ancestors very sagely prohibited amputation until the barrier was established, and amputated without delay when it was, rightly appreciating the nature of the case, and knowing that the system, which had not power to stop a destroying inflammation, à fortiori, had not power to initiate a healing one, and consequently that the same mischief would fall upon the stump. We have not advanced, and cannot improve upon this sound and practical pathological axiom.

"Gangrenous inflammation is characterised by the moist state of the part, owing to the atony of the exhalant vessels; a peculiar dusky livor, and emphysematous swelling, with sanious vesicles here and there appearing; the pain, often excruciating; and an undefined and irregular margin from the deficiency or tardy formation of the lymph barrier. To these, as disorganization advances, may be added the cadaveric fetor and the loss of temperature compared with that of the neighbouring sound parts.

"The fever is either the mild inflammatory, or the typhoid in its commencement; the first speedily lapses into the second, marked by frequent sinking and call for support; livid and cadaverous complexion, deficient alvine and urinary secretions, thirst, brown, or black furred tongue, hiccup, cold clammy skin, anxiety, and propensity to rambling, or muttering delirium. The pulse, yielding to the stimulus applied, is an artificial one, until the vital powers are so far sunk as to be no longer responsive to it; but even with the swell communicated by ammonia, wine, and opium, it has no real resistance, being easily compressible; and naturally quick, thready, and powerless." (P. 165.)

Mr. Travers then alludes to the erroneous ideas prevalent on gangrene, which he attributes to the confusion of gangrene and gangrenous inflammation; to the latter ensuing occasionally upon the former; to the constitutional condition being regarded as a secondary state, and seldom as the primary one; and to the remarkable variety of results,—in one case the establishment of mortification being equivalent to death, and in another a state comparatively free from danger; one man parting with both his feet with scarcely any illness, and another dying in three days after the appearance of sanious vesicles upon the instep, with all the local and constitutional symptoms of rapid mortification. This discrepancy, says Mr. Travers, is reconciled by reference to the constitution. If it is sound, the effect of the mechanical death of a part is to rouse it to a preservative action,—the adhesive; if it be infirm, the gangrene spreads. If the part be previously inflamed, particularly by erysipelas, the continued local or general excitement will determine the gangrenous inflammation; but, if recent and not very extensive, (both when spontaneous, and much more after injury,) it may be arrested, and the ulcer healed by supporting the patient's powers. The danger is greater according to the part; greatest if it interferes with a vital function, more dangerous in the trunk than the limbs; but the great difference is according to the constitution,—whether the patient be sound or diseased, strong or weak, young or aged; the subject of present or recent fever, or other constitutional ailment, or the contrary. Thus, Mr. Travers has seen the application of a small caustic issue to a scrofulous tumour, on the neck of a boy, followed by gangrenous inflammation, and death on the third day; and the application of six leeches to a small, painful, but uninflamed tumour in a young woman's breast, succeeded by gangrenous inflammation of the whole mamma in twenty-four hours, and death on the next day. Frostbite of the foot is often unattended with any serious constitutional disturbance, but in old starved subjects it is fatal; and, if spontaneous, the result of a worn-out system or diseased heart and vessels, it is almost always fatal. The slough of the penis is rarely fatal; that of the nates very generally: the latter is part of the trunk. From the impoverished systems of free livers and hard drinkers, the gangrenous inflammation may stop on one side, extend in another, and then overshoot the boundary line, the newly formed granulations alternately sloughing; and thus entire limbs are affected from a seemingly insignificant beginning.

These remarks are so much to the point, and contain the results of such just and discriminative observation, that they throw much light on a very obscure subject. They are illustrated by cases, and followed by some pertinent remarks on the treatment of gangrenous inflammation.

"A mistake often committed by practitioners is an immediate recourse to stimulants: great additional mischief sometimes results from it. I have often seen patients restored under a gentle course of carminative aperients, with occasional salines, and a diaphoretic opiate at night, where severe sloughing had already taken place; the inflammation of the surrounding part has abated and the progress of the destruction has been arrested, and then tonics and wine have been administered with the happiest effect." (P. 200.)

Where there has been acute pain, a full habit, or flushed face, Mr. Travers has bled once with advantage. For the fever he has given salines, gentle aperients, and a full opiate at bedtime; mercurials, if the secretions require it. There is a period, often an early one, at which ammonia may be given, from five to ten grains for a dose, with the best effects. In these cases, quinine with sulphuric acid is useful. Opium, however, is the most beneficial drug, both in allaying irritation and in supporting the powers of the constitution. For the local treatment, leeches in large numbers, when venesection is out of the question, particularly when it follows severe contusion.

"The linseed, and especially the yeast or charcoal poultice, with poppy and chamomile fomentations, or opium suspended in mucilage and water; the chlorates of lime or soda, half an ounce or more of the solution to half a pint of water; the diluted nitric acid; the lunar caustic lotion; the black wash, with or without opium, are the best applications in my experience; these may be at first covered by the poultice, and afterwards by cerate. In cases of inaction, turpentine and olive oil, the benzoic tincture, the peruvian balsam, and the compound elemi ointment, are useful dressings; but the strong nitric acid most quickly and beneficially changes the surface. A lotion composed of three drachms of the chloride of lime, one drachm of caustic potass, and twelve ounces of distilled water, very speedily removes those dense crusts of ash-coloured slough which adhere so tenaciously to the bed of gangrenous ulcers." (P. 203.)

The last chapter on the subject of reflected irritation contains a recapitulation of Mr. Travers's views, explanations and justifications of his terms, and observations, particularly on the state of pathology, which are often very just and comprehensive. He has explained with force and truth the errors produced by morbid anatomy holding the first, and almost the only, place in the mind of the medical enquirer. Effects are thus substituted for causes, and the laws of physics for the laws of life; and the cause of death is confounded with the cause of disease. But, Mr. Travers adds,

"I venerate that study for its power of confirming or correcting the opinions of the cautious and intelligent student of living nature; of shedding light where darkness reigned supreme; developing and enabling us to connect and compare the characters of morbid changes with the phænomena of disease during life, and thus to establish inferences, and gradually to arrive at a knowledge of the principles and laws which regulate morbid actions." (P. 212.)

The whole chapter is well worthy of attentive perusal, as it contains the reflections of one who has seen the growth (almost from its commencement) of the school of pathological anatomists, and has attentively studied its effects. We regret, however, that Mr. Travers has indulged in a sneer at the attempts of pathologists to investigate morbid changes by the aid of the microscope. The observations of Gendrin, Kattenbrunner, &c. are surely worthy of a more candid comment than "these things, and a thousand others not less extraordinary, may be; yet, if knowledge has no bounds for our senses, its advancement must keep some measure with our understandings, or it ceases to be useful." (P. 222.) Such principles would exalt the reason to that fatal pre-eminence which it held in scientific enquiries before the time of Bacon: "Subtilitas naturæ subtilitatem sensûs et intellectûs multis partibus superat." Hence we are bound to use all those helps to the senses which modern discoveries may supply, and to look to such investigations for actual additions to our knowledge.

Let the most incredulous examine the boiled crystalline lens of a codfish with a modern microscope, and he will not wonder at any other microscopic discovery. We are convinced that John Hunter, were he now living, would be pursuing his investigations on inflammation with these helps. He, who would stand over an insect, dissecting it with a pin for four consecutive hours, would certainly never have thought attention to the microscopic detail of morbid processes beneath him. If the French investigators have not looked beyond the changes they have described, their theories may be bad, but the facts remain as materials for more philosophical induction. Into this want of candour Mr. Travers has been led by rather too exclusively attaching himself to the valuable pathological principles on which his whole work is founded; and, although he deprecates exclusiveness in others, we think it leads him too generally to direct his attention to states of the whole constitution, rather to the neglect of local organic disease. There are some valuable cases scattered throughout the volume, in which death after operations, secondary inflammations, &c., seemed to depend on the mere exhaustion of the constitution from suppuration, or some external inflammation not involving a vital organ; but the surgeon should bear in mind that these cases are rare, and that, as a general rule, it will be safer for him to be guided in his prognosis by the healthy or diseased state of those organs essential to life. It strikes us that Mr. Travers, on the same grounds, passes over too lightly both the influence exerted by the digestive organs in inducing these diseased states, and the necessity of directing our remedies in this direction. The stomach is the "centre of sympathies," and obviously the great inlet of disease; and, to use the forcible simile of a vigorous writer, who a century ago enforced what we now call Abernethian doctrines, "the cure must begin where the evil began; and must be communicated thence to the rest of the system, as a ropemaker begins the twist at one end of the rope, and communicates it to all the other parts."

We have already alluded to one evil resulting from the exclusive attention paid to diseased products, the correction of which Mr. Travers's writings are calculated to effect: they will also tend to dissipate another resulting from the same cause, and shown in a disposition to under-rate reasoning upon the facts thus assiduously collected. Mr. Travers is one of those surgeons who admit that the most enlightened practice must derive its value from study and reasoning. He pursues his profession with enlarged views, and is evidently not content with the knowledge to be derived from mere experience. It is by such cultivators of surgery that it is ever best improved. They do not merely succeed in establishing points of practice, but direct attention to the principles of the science.

In concluding our remarks on the first part of this work, we willingly concede to our author the merit of earnestly endeavouring to direct the attention of surgeons to the best method by which a knowledge of the diseases they have to treat can be obtained. His object is to elevate surgery, and to cultivate it on scientific principles. He has laboured to elucidate some of the most important and difficult points connected with its pathology; and if, in the prosecution of his object, he has occasionally failed in producing conviction by his theories, we are not, on that account, to pay the less attention to the great quantity of valuable matter which he has laid before us.

